# An agile, data‐driven approach for target selection in rTMS therapy for anxiety symptoms: Proof of concept and preliminary data for two novel targets

**DOI:** 10.1002/brb3.2914

**Published:** 2023-03-22

**Authors:** Isabella M. Young, Hugh M. Taylor, Peter J. Nicholas, Alana Mackenzie, Onur Tanglay, Nicholas B. Dadario, Karol Osipowicz, Ethan Davis, Stephane Doyen, Charles Teo, Michael E. Sughrue

**Affiliations:** ^1^ Omniscient Neurotechnology Sydney New South Wales Australia; ^2^ Cingulum Health Sydney New South Wales Australia; ^3^ Rutgers Robert Wood Johnson School of Medicine New Brunswick New Jersey

**Keywords:** anxiety, brain stimulation, repetitive transcranial magnetic stimulation, treatment

## Abstract

**Introduction:**

Data‐driven approaches to transcranial magnetic stimulation (TMS) might yield more consistent and symptom‐specific results based on individualized functional connectivity analyses compared to previous traditional approaches due to more precise targeting. We provide a proof of concept for an agile target selection paradigm based on using connectomic methods that can be used to detect patient‐specific abnormal functional connectivity, guide treatment aimed at the most abnormal regions, and optimize the rapid development of new hypotheses for future study.

**Methods:**

We used the resting‐state functional MRI data of 28 patients with medically refractory generalized anxiety disorder to perform agile target selection based on abnormal functional connectivity patterns between the Default Mode Network (DMN) and Central Executive Network (CEN). The most abnormal areas of connectivity within these regions were selected for subsequent targeted TMS treatment by a machine learning based on an anomalous functional connectivity detection matrix. Areas with mostly hyperconnectivity were stimulated with continuous theta burst stimulation and the converse with intermittent theta burst stimulation. An image‐guided accelerated theta burst stimulation paradigm was used for treatment.

**Results:**

Areas 8Av and PGs demonstrated consistent abnormalities, particularly in the left hemisphere. Significant improvements were demonstrated in anxiety symptoms, and few, minor complications were reported (fatigue (*n* = 2) and headache (*n* = 1)).

**Conclusions:**

Our study suggests that a left‐lateralized DMN is likely the primary functional network disturbed in anxiety‐related disorders, which can be improved by identifying and targeting abnormal regions with a rapid, data‐driven, agile aTBS treatment on an individualized basis.

## Graphical abstract

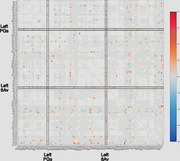

## 1 INTRODUCTION

While it is increasingly accepted that transcranial magnetic stimulation (TMS) is a safe and effective option for many patients with mental illnesses, most notably patients with treatment‐resistant major depression (MDD); patient responses to TMS treatments are highly variable (Berlim et al., 2017; Blumberger et al., 2018; Jannati et al., 2017). Many patients do not respond to standard approaches, such as the traditional craniometric targeting of the dorsolateral prefrontal cortex (DLPFC), and patients who do respond do not show resolution of all symptoms (Fox et al., 2012, 2013; Johnson et al., 2013; Weigand et al., 2018). One possible explanation for these differential patterns of response may be that these disorders demonstrate significant heterogeneity between patients, likely reflected in heterogeneous abnormalities of functional connectivity (Clementz et al., 2015; Drysdale et al., 2017; Nestler & Hyman, 2010). Indeed, given the often‐diverse range of connectivity anomalies seen in patients with mental illnesses, it may be that different symptoms of a disorder might each result from different, unique network abnormalities that need to be addressed individually rather than in a singular diagnosis protocol (i.e., the transdiagnostic hypothesis of mental illness can be extended to connectomics) (Drysdale et al., 2017; Siddiqi et al., 2020). For instance, MDD includes symptoms of both anxiety and anhedonia, but these two symptoms localize to different brain circuits and respond best to different TMS targets (Siddiqi et al., 2020). Given that patient‐specific differences in underlying cortical networks influence the location of TMS stimulation targets, current, standard of practice TMS targeting based on general scalp measurements can provide limited benefits in patients with a wide symptom profile. Data‐driven approaches can provide consistent, empirical models of symptom localization based on functional connectivity analyses that can be applied for individualized TMS targeting.

In patients with depression, data on group‐average connectivity (“group maps”) can identify coordinates that may optimize antidepressant responses following TMS treatment (Fox et al., 2012). However, limitations and difficulty with the technology and models utilized in single‐subject analyses, lead to variable ideographic results that are difficult to translate clinically (Herwig et al., 2003). While the feasibility of individualized connectivity‐based targeting of the left DLPFC has been illustrated in patients with depression, similar individualized methods have not been demonstrated for patients with GAD (Fox et al., 2013). Given the significant heterogeneity in individual functional connectivity in patients with GAD as well as the heterogeneity of results in different rTMS trials utilizing group or single‐subject data for TMS target selections, the demonstration of a feasible single‐subject, data‐driven, target selection method for rTMS in GAD patients could inform larger clinical trial designs in the future.

While this makes intuitive sense, most clinical TMS research involves the use of randomized controlled trials (RCT) as a method for hypothesis testing. This remains the gold standard for validating a clinical hypothesis, there are some limitations to such an approach, notably that it is slow, resource‐intensive, and inflexibly ties researchers to an a priori hypothesis (Bothwell et al., 2016). Additionally, for appropriately powered clinical trials and subsequent reviews, it requires significant case numbers of both treatment and sham patients, and requires replication when investigating different treatment locations or protocols. Not only is this costly to undergo, but it requires hundreds, if not thousands of individuals receiving sham treatment. We do not suggest that implementing RCTs is not incredibly useful and should be the gold standard, however we suggest that given the vast investment of resources and time they involve, smaller studies investigating treatments that appear successful paired with the idea that a more individualized approach may be the best way forward, that smaller cohort studies can provide important insights that should not be overlooked.

In this report, we provide a proof of concept for a different approach, namely using an agile target selection paradigm based on patient‐specific connectomic methods to detect abnormal functional connectivity, guided treatment aimed at the most abnormal regions, and the rapid development of new hypotheses for future study. Using this individualized approach in a cohort of patients with treatment‐resistant generalized anxiety disorder (GAD), we identify two regions, left 8 Av and left PGs, which show consistent functional connectivity abnormalities between patients, and which when treated, showed very promising results which should be considered in future, larger clinical trials. Moreover, given even limited success, we demonstrate the ability to utilize agile‐connectomics‐based‐individualized‐targeting methodology for rapid and personalized treatment development.

## 2 MATERIALS AND METHODS

### 2.1 Participants

Patients (*n* = 28) were included in this study if they had a medically refractory anxiety disorder during a 12‐month period in our outpatient TMS clinic. Patients were not excluded if they had comorbid mental illnesses or were taking prescribed medications (real‐world conditions whereby patients were instructed to continue their medication dose throughout treatment).Similarly, participants were included even if their GAD‐7 scores did not meet the cut‐off for generalized anxiety disorder so long as their prescribing practitioners had evidence of a history of medically refractory anxiety with persistent and ongoing symptoms at the time of prescription. All participants gave informed consent after being informed of the fMRI based agile‐targeting approach and its difference from standard approaches.

### 2.2 Diffusion‐weighted and Resting‐State Functional MRI acquisition

A week before treatment, all patients underwent neuroimaging. Imaging studies were performed on a Phillips 3T Achieva. Diffusion‐weighted imaging was performed with the following acquisition parameters: 2 mm × 2 mm × 2 mm voxels, FOV = 25.6 cm, matrix = 128 mm × 128 mm, slice thickness = 2.0 mm, one nonzero *b*‐value of *b* = 1000, 40 directions, gap = 0.0 mm. A resting‐state functional MRI (rsfMRI) was acquired as a T2‐star EPI sequence, with 3 × 3 × 3‐mm voxels, 128 volumes/run, a TE = 27 ms, a TR = 2.8 s, a field of view – 256 mm, a flip angle = 90° and an 8‐min total run time.

### 2.3 Image processing pipeline

Resting‐state and diffusion preprocessing was performed using the Omniscient Infinitome software (Sydney, Australia) (Omniscient Neurotechnology, 2020), which is a pipeline for machine learning–based brain image processing pipelines. The specific details for the algorithms are described below. In short, the pipeline is cloud‐based which utilizes microservices to run custom‐written Python‐based scripts in a Kubernetes's serverless framework. The viewing user interface (UI) platform runs in Angular JS inside a Chrome browser, and target selection was made in this UI.

### 2.4 Diffusion tractography preprocessing steps

The DT images are processed using standard processing steps which specifically include the following steps: (1) the diffusion image is resliced to ensure isotropic voxels, (2) motion correction is performed using a rigid body alignment, (3) slices with excess movement (defined as DVARS > 2 sigmas from the mean slice) are eliminated, (4) the T1 image is skull stripped using a convolutional neural net (CNN), this is inverted and aligned to the DT image using a rigid alignment, which is then used as a mask to skull strip the DT, (5) gradient distortion correction is performed using a diffeomorphic warping method which aims to locally similarize the DT and T1 images, (6) eddy current correction is performed, (7) fiber response function is estimated and the diffusion tensors are calculated using constrained spherical deconvolution, (7) deterministic tractography is performed with random seeding, usually creating about 300,000 streamlines per brain.

### 2.5 Creation of a personalized brain map using machine learning–based parcellations

The Infinitome Neuroscience Platform (Sydney, Australia) creates a machine learning–based, subject‐specific version of the Human Connectome Project Multi‐Modal Parcellation version 1.0 (HCP) atlas based on diffusion tractography structural connectivity (Glasser et al., 2016). This method has been described by Doyen et al. (2022). This novel method was created by training a machine learning model on 200 normal subjects by first processing T1 and DT images as above and has been described in our previous work (Doyen et al., 2022). An HCP atlas in NIFTI Montreal Neurological Institute (MNI) space was then warped onto each brain and the structural connectivity was calculated between every pair of this atlas and a set of Region of Interest (ROIs) containing 8 subcortical structures per hemisphere and the brainstem based on the streamlines which terminated within an ROI. These feature vectors for each region were then used as a training set and the data were modeled using the XGBoost method.

This model is then applied to the new subject by first, warping the HCP atlas to the new brain and collecting a set of feature vectors of the connectivity of each voxel. The feature vectors are then used to determine if each voxel belongs to a parcellation or region or not and if so to assign the voxel to that parcellation. This creates a version of the HCP atlas, with subcortical components, which is not dependent on brain shape or pathologic distortion, and which is specific for this subject, but comparable between subjects.

### 2.6 rsfMRI preprocessing

The rsfMRI images are processed using standard processing steps which specifically include the following: (1) motion correction is performed on the T1 and BOLD images using a rigid body alignment; (2) slices with excess movement (defined as DVARS > 2 sigma from the mean slice) are eliminated; (3) the T1 image is skull stripped using a convolutional neural net (CNN), this is inverted and aligned to the resting‐state bold image using a rigid alignment which is then used as a mask to skull strip the rsfMRI image; (4) slice time correction is performed; (5) global intensity normalization is performed; (6) gradient distortion correction is performed using a diffeomorphic warping method which aims to locally similarize the rsfMRI and T1 images; (7) high variance confounds are calculated using the CompCor method (Behzadi et al., 2007); these confound as well as motion confounds are regressed out of the rsfMRI image and the linear and quadratic signals are detrended. Note this method does not perform global signal regression. (8) Spatial smoothing is performed using a 4 mm full width at half maximum Gaussian kernel.

### 2.7 rsfMRI correlation and anomaly detection

The personalized atlas created in previous steps is registered to the T1 image and localized to the gray matter regions. Thus, it is ideally positioned for extracting an average BOLD time series from all 377 areas (180 parcellations × 2 hemispheres, plus 17 subcortical structures). This yields 142,129 correlations. Outlier detection using a tangent space functional correlation matrix was performed by comparing the results with a subset of 200 normal subject rsfMRI samples in whom a tangent space connectivity transformation was performed to determine the range of normal correlations for each functional connectivity pair in the matrix. Abnormal connectivity was determined as a 3‐sigma outlier for that correlation, after excluding the highest variance 1/3 of pairs, to further reduce the false discovery rate.

The assignment of parcellations to various large‐scale brain networks was based on several previous coordinate‐based meta‐analyses and matching the HCP parcellations to the coordinates of the activation likelihood estimation (ALE) in MNI space, which has been previously published (or in review presently) by our group (Milton et al., 2021; Sandhu et al., 2021). Notably, a significant amount of the data on HCP parcellations illustrated by Akiki and Abdallah (2019) contributed to our parcel‐network classification.

### 2.8 Method of agile target selection

**FIGURE 1:**
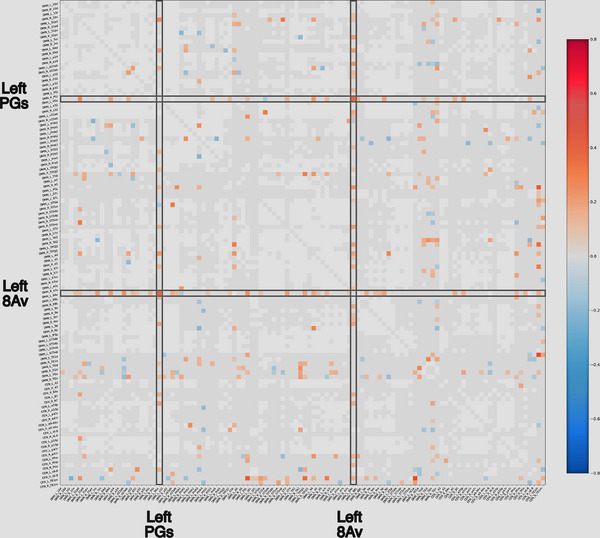
Anomalous functional connectivity matrices. Anomalous functional connectivity matrices for the Default Mode Network (DMN) and Central Executive Network (CEN) based on functional connectivity data for two patients in our cohort. Targets were generally represented by columns demonstrating multiple anomalies in functional connectivity with intra‐ and internetwork connectivity. Light gray squares are considered to be functioning normally when compared to a normal dataset, and white squares are areas that are highly variable, and are thus excluded from the analysis. The chosen targets for treatment are highlighted by gray boxes. If an area showed mostly hyperconnectivity with other areas, we used continuous theta burst stimulation on the hypothesis that it generally induces cortical depression and we chose intermittent theta burst stimulation for areas which generally displayed a majority of anomalies which were hypoconnected to other parts of these networks. The prescribed treatment protocol for this patient was continuous theta burst stimulation of left 8Av and left PGs.

We approached individual target selection by allowing the functional connectivity data to guide the selection of targets for TMS treatment with a minimal set of a priori constraints on target selection. We started with the hypothesis that functional connectivity abnormalities between parcellations in the Default Mode Network (DMN), central executive network (CEN), and/or salience network (both inside of the network and/or outside of network) underlie common abnormalities seen in mental illnesses, including generalized anxiety disorder as demonstrated by multiple lines of evidence (Menon, 2011). We thus looked at the anomalous functional connectivity matrices produced for these networks (Figure 1), looking for evidence of abnormal functional connectivity. Targets were generally represented by columns demonstrating multiple anomalies in functional connectivity with other members of these three networks. These columns indicate parcellations that show outlier functional connectivity with multiple other regions of the DMN, CEN, and/or salience networks.

Importantly, we made *no* subjective, conscious efforts to guide any patient to a standardized target approach. Instead, these decisions were purely based on the objective functional connectivity data in which connectivity anomalies outside of 3 sigmas of the normal range from 200 healthy rsfMRI data were chosen as TMS targets.

We generally aimed to identify two to three targets. Whenever present, we usually put a slight priority on identifying areas in the DLPFC, based on prior work in the field as a biasing factor (Diefenbach et al., 2016; Siddiqi et al., 2020).

We used the anomalous functional connectivity algorithm to guide the selection of intermittent theta burst stimulation (iTBS) or continuous theta burst stimulation (cTBS) protocols. In short, if an area showed mostly hyperconnectivity with other areas, we used cTBS on the hypothesis that it generally induces cortical depression (Huang et al., 2005). The converse was also true, that we chose iTBS for areas which generally displayed a majority of anomalies which were hypoconnected to other parts of these networks (Huang et al., 2005).

### 2.9 TMS treatment

The treatment paradigm used was an accelerated theta burst stimulation (aTBS) protocol, consisting of 5 image‐guided TBS treatment sessions per day for 5 days, which started hourly (Rashid & Calhoun, 2020). As demonstrated in Table 1, all of the targets listed for each subject were consecutively stimulated in each treatment session at 80% of the resting motor threshold. iTBS was performed as bursts of 3‐pulse 50‐Hz bursts given every 200 ms at (5 Hz) for 40 trains, with an intertrain interval of 6.3 s, for a total of 1200 pulses. cTBS was performed as one train of 600 stimuli applied in 3‐pulse 50‐Hz bursts given every 200 ms (5 Hz), for a total of 1800 pulses. All TBS sessions were completed using a Magventure MagPro X100 TMS machine with a butterfly cool coil (Alfaretta, USA). Each patient's individualized treatment protocol can be seen in Table 1.

**TABLE 1:** Patient characteristics

					Anxiety score (GAD‐7)			
Participant ID	Age	Gender	Target	Stimulation protocol	Pretreatment	Posttreatment	1 Week posttreatment	1 Month posttreatment
1	57	Male	Left 8Av	iTBS	15	12	3	2
			Right 43	cTBS				
2	21	Male	Left PGs	cTBS	10	5	4	4
			Left 8Av	cTBS				
3	29	Female	Left PGs	cTBS	21	21	18	19
			Right PFm	iTBS				
4	32	Male	Left 8Av	cTBS	8	3	–	–
			Left PGs	cTBS				
5	18	Female	Left 8Av	cTBS	19	12	–	–
			Left PGs	iTBS				
6	44	Male	Left 46	iTBS	2	2	0	0
			Right 46	iTBS				
7	51	Male	Left 8Av	cTBS	17	8	6	–
			Left PFm	cTBS				
8	31	Male	Left 8Av	cTBS	2	6	–	–
			Left PFm	cTBS				
			Left 46	cTBS				
9	49	Male	Right s6‐8	cTBS	3	4	–	5
			Left 8Av	cTBS				
10	23	Female	Right s6‐8	cTBS	9	8	4	2
			Left PGs	cTBS				
11	18	Male	Left 8Av	cTBS	12	13	–	–
			Left PGs	cTBS				
			Right TE1m	cTBS				
12	46	Female	Left 8Av	cTBS	21	3	–	6
			Left PGs	cTBS				
13	30	Female	Left 8Av	cTBS	7	2	–	3
			Right TE1m	iTBS				
14	66	Male	Left 8Av	cTBS	4	5	–	–
			Right TE1m	iTBS				
15	49	Male	Left 46	cTBS	11	6	2	2
			Left 8Av	cTBS				
16	31	Male	Right s6‐8	cTBS	1	2	–	–
			Left PFm	cTBS				
			Left PGs	cTBS				
17	34	Male	Left 8Av	cTBS	0	6	1	0
			Left PGs	cTBS				
18	55	Male	Left 8Av	cTBS	15	14	9	–
			Left PGs	cTBS				
19	20	Male	Left 8Av	cTBS	10	15	12	5
			Left PGs	cTBS				
			Left PFm	cTBS				
20	18	Female	Left 8Av	cTBS	18	12	6	–
			Left 6ma	cTBS				
21	20	Male	Left 8Av	cTBS	9	10	–	–
			Left PGs	cTBS				
22	39	Male	Left 8Av	cTBS	12	5	7	5
			Left PGs	cTBS				
23	71	Female	Left 8Av	cTBS	8	5	–	13
			Left PGs	iTBS				
24	38	Male	Left 8Av	iTBS	20	1	–	–
			Right PGs	cTBS				
25	49	Female	Left 8Av	iTBS	17	4	–	–
			Left PGs	cTBS				
26	43	Female	Left 8Av	cTBS	21	21	–	–
			Left PGs	cTBS				
			Right 8Av	cTBS				
27	40	Male	Left 8Av	iTBS	19	4	–	–
			Left PGs	iTBS				
28	48	Female	Left 8Av	cTBS	8	3	–	–
			Left PGs	iTBS				

The targets were exported from the Infinitome Neuroscience Platform (Sydney, Australia) and uploaded as NIFTI volumetric objects, coregistered to their T1 file, into the Localite (Bonn, Germany) image guidance platform. The Localite navigation system was used to register the patient's anatomy to their T1 image using surface point tracing. The target centroid was chosen as the area to center the TMS probe over the target.

### 2.10 Primary outcome measures

The primary outcome measure was Generalized Anxiety Disorder 7‐item (GAD‐7) (Fusar‐Poli et al., 2019). All participants completed these self‐report questionnaires before initiating treatment and following their final treatment and some participants completed 1‐week posttreatment and 1‐month posttreatment follow‐up scores.

### 2.11 Statistical methods

Differences between pre‐ and posttreatment scores for each outcome measure were calculated using a paired *t*‐test and repeated‐measures ANOVA. In order to maintain as much data as possible for the analyses, missing observations were replaced with the sample mean (for that time‐point). We acknowledge that this artificially inflates our sample size; therefore, we repeated the analyses excluding subjects who did not have all of the compared data. All continuous measures are reported as mean ± standard deviation.

## 3 RESULTS

### 3.1 Participant characteristics

Twenty‐eight participants, *n* = 18 men and *n* = 10 women (mean age = 38.2 ± 14.8), completed the course of treatment. Comorbidities were prevalent in 85.7% of the cohort, wherein 18 participants were experiencing comorbid MDD, 3 with posttraumatic stress disorder, 3 with obsessive compulsive disorder, and 1 case of chronic pain and 1 case of borderline personality disorder. Some of the participants had multiple comorbidities. Patient information, including targets, treatment protocol, and GAD‐7 scores are listed in Table 1. The incidental sampling of follow‐up assessments led to a high rate of subject attrition, as the research was secondary to clinical care. Only 9 participants completed all follow‐up metrics, with an additional 9 completing 3 of the 4 follow‐up metrics.

### 3.2 Consistent abnormal functional connectivity in anxiety patients

8Av and PGs demonstrated consistent abnormalities, particularly in the left hemisphere, among the anxiety patients. Twenty‐four participants had left 8Av as a target (83% cTBS). Left PGs was chosen as a target in 18 participants (78% cTBS). Thirteen participants had a treatment prescription with both left 8Av and left PGs as targets. Only one participant did not have either area 8Av or PGs in either hemisphere chosen as a treatment target.

### 3.3 Anxiety improves in these patients

**FIGURE 2:**
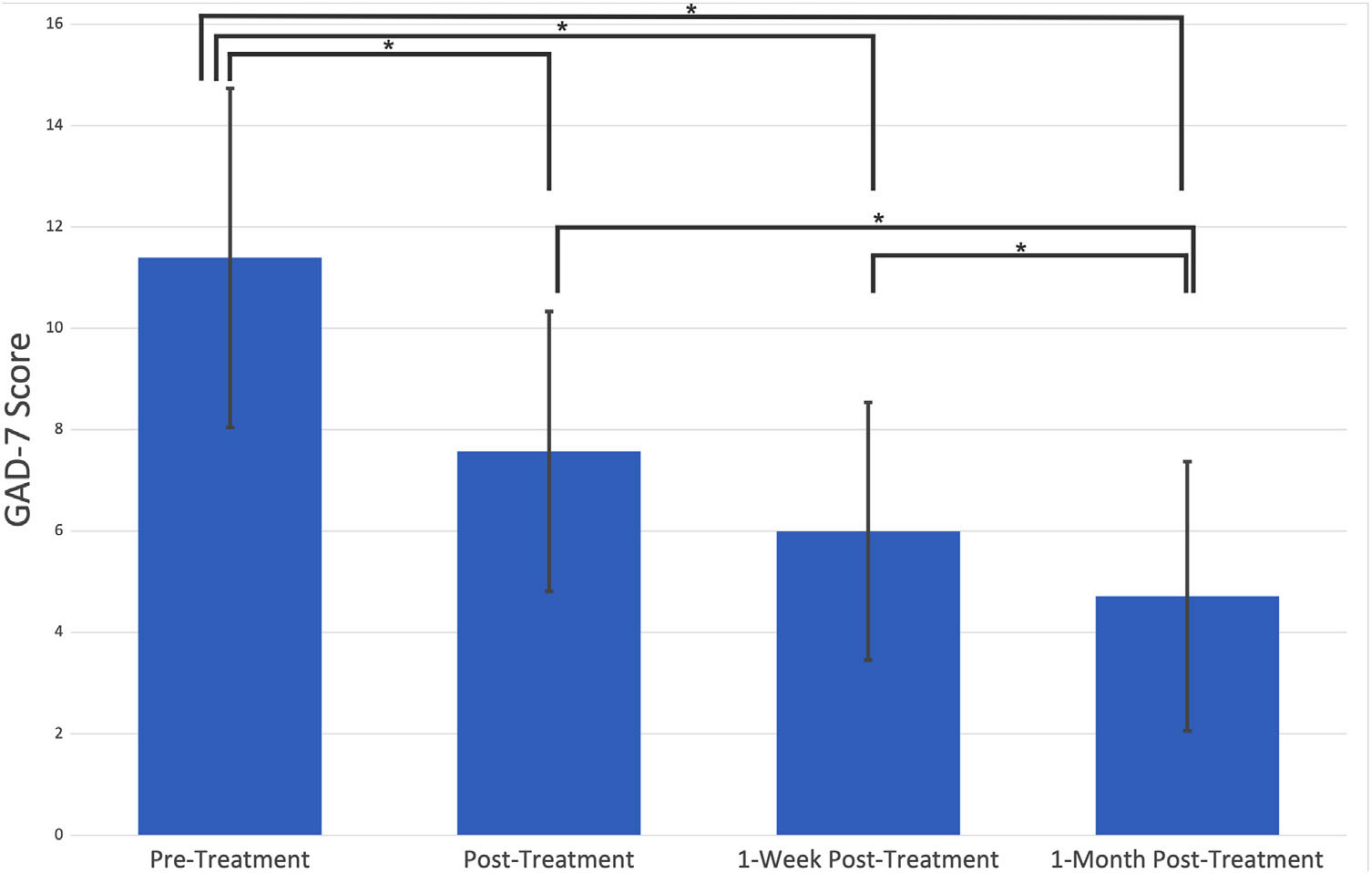
Average GAD‐7 scores of participants before and after treatment. Participants completed the GAD‐7 immediately prior to, and following Transcranial Magnetic Stimulations using the agile‐targeting approach. Additional follow‐up metrics of the GAD‐7 were taken 1 week and 1 month after their treatment completion.

As seen in Figure 2, on average anxiety symptoms improved immediately posttreatment in the cohort. Patient scores on the GAD‐7 scale significantly decreased immediately at the end of treatment (*M* = 7.57, SD = 5.52), demonstrating a significant improvement in anxiety symptoms (*t*(27) = 3.15, *p* = .004). This reduction in anxiety persisted, with both 1‐week (*M* = 6.0, SD = 5.08, *t*(27) = 4.61, *p* = .00009) and 1‐month (*M* = 4.71, SD = 5.31) posttreatment scores being significantly decreased from pretreatment scores (*t*(27) = 5.69, *p* = .000005). Overall, there was a statistically significant mean decrease of anxiety scores across time, *F*(3, 78) = 5.08, *p* = .0029. We repeated the above analyses excluding all participants with missing data. There was once again a significant decrease in pre‐ to 1‐week post‐GAD‐7 scores (*t*(11) = = 4.16, *p* = .0007); a significant decrease in pre‐ to 1‐month post‐GAD‐7 scores (t(13) = 3.17, *p* = .007); and a significant mean decrease of anxiety scores across time (*F*(2, 24) = 2.2, *p* = .04).

### 3.4 Complications

Two patients experienced fatigue after the third day of treatment until their treatment course ended. One patient had a dull headache after the first day of treatment. The remaining patients did not report any side‐effects.

## 4 DISCUSSION

An increasing body of evidence suggests that mental illness classifications group together a heterogeneous collection of diverse pathophysiologic states based on partial symptom overlap (Drysdale et al., 2017; Fusar‐Poli et al., 2019; Krueger & Eaton, 2015). Importantly, a large number of studies also suggest that functional connectivity measures can provide insights into the connectivity differences between individuals and provide a method toward understanding the mechanism of this heterogeneity (Cole et al., 2020; Fox et al., 2013; Rashid & Calhoun, 2020). Therefore, an individualized, connectomic approach can guide therapies toward pathophysiologic signature profiles of patient‐specific symptoms which transcend traditional singular diagnostic categories to optimize clinical outcomes (Siddiqi et al., 2020).

The present report provides preliminary evidence suggesting that using a connectomic‐ approach based on resting‐state functional connectivity (rsfMRI) can identify consistent patterns of connectivity disturbances in patients with medically refractory generalized anxiety (GAD). This empirical, data‐driven information can be specifically modulated with image‐guided noninvasive brain stimulation demonstrating favorable therapeutic improvement in anxiety symptoms. Interestingly, these patients show relatively consistent functional anomaly patterns in two parcellations of the left Default Mode Network (DMN), 8Av, and PGs, which were our most common targets in the current study. Note, we initially approached this sample in a target naïve fashion to avoid bias, and mainly directed our target selection strategy by identifying anomalies inside of the DMN, CEN, and their cross‐network connectivity through a machine learning approach with an anomalous functional connectivity matrix. These promising preliminary results highlight the potential for using connectomic‐based approaches to find individualized symptom‐specific targets in GAD which can be applied for other symptom clusters related to different mental illnesses.

### 4.1 The potential for an open‐label paradigm for TMS targeting discovery research

The majority of clinical TMS research has focused around the randomized controlled trial (RCT). This kind of study paradigm involves evaluating an a priori hypothesis regarding the target, stimulation parameters, and inclusion and exclusion criteria. This phase was critical in establishing TMS as an effective approach for diseases such as major depressive disorder (MDD) (Berlim et al., 2017). However, given the idea of treating mental illness with TMS has become less controversial with an increasing body of evidence and experience for the safety of this technique over recent years, it is worth revisiting the idea of whether an RCT is the best approach for hypothesis exploration (Berlim et al., 2017; Duprat et al., 2016). Given the known heterogeneity of the types of diseases being treated with TMS, it is possible that more specific subtyping may allow for at least some patients to receive more effective treatment with different targets, possibly with different protocols, and that subtype‐specific trials may yield more consistent results (Fox et al., 2013; Siddiqi et al., 2020).

A similar study by White and Tavakoli (2015) demonstrated high rates of remission among a cohort of MDD with comorbid anxiety. They demonstrated a higher remission rate than that of our study, although there are a number of differentiating factors that could be contributing to this. An interesting point was that they completed more treatments over time than our clinical workflow, which could indicate that patients with anxiety, particularly those with comorbidities, may require additional sessions in order to reach remission. Additionally, their study also elucidates the potential benefit of bilateral treatments, of which some of our participants had as well. These hypotheses are worthwhile additional research and further demonstrate the benefit of different study designs to generate interesting hypotheses worthy of exploration.

It is also worth noting that the placebo effect may be enhanced with the use of medical devices, compared to pharmacotherapy. This is thought to be due to several factors associated with the treatment, including its association with sophisticated technology, the placement of a device being placed over the head, and increasing media coverage of its innovative use (Burke et al., 2019). While several studies have noted this enhanced placebo effect (Razza et al., 2018), it has not been extensively studied. Given our study was a preliminary proof‐of‐concept study, we did not have a control condition, making it difficult to comment on the role of the placebo effect. However, while several trials utilizing rTMS, most notably for depression, have demonstrated benefits, more structured, larger trials with longer follow‐up are needed to address treatment effects. This includes addressing questions such as whether sham rTMS relying on the placebo effect itself could be a therapeutic option for some patients.

### 4.2 Why is the left side the abnormal side?

It was a bit surprising to us that the functional anomalies were so frequently on the left side in these patients, but the data was strikingly asymmetric in this direction. Given previous literature with right‐sided anxiety treatments, we were surprised at how consistent the left‐sided anomalies were in these patients, and how consistent our results were with treating these areas with cTBS (Bystritsky et al., 2008; Diefenbach et al., 2016; Dilkov et al., 2017; Sagliano et al., 2019; White & Tavakoli, 2015).

An answer may lie in subtle differences in the network affiliation of different parcellations between the two hemispheres as seen in connectomic studies of rsfMRI data in normal individuals (Akiki & Abdallah, 2019). Specifically, there are few asymmetries in these maps, usually, a parcellation which is part of the extended CEN or DMN on the right side has the same affiliation on the left (Akiki & Abdallah, 2019). However, we noted that the asymmetries are particularly interesting, for example, area 44 and area 55b (speech/language areas) are DMN parcellations on the left (consistent with the idea that the DMN plays a role in language production) and CEN parcellations on the right (Akiki & Abdallah, 2019; Sheets et al., 2021). A similar pattern has been shown with 8Av and PGs, which on the left are DMN affiliated, and on the right are CEN affiliated, a pattern not repeated in their immediate neighbors (Akiki & Abdallah, 2019). This striking finding provides a possible mechanism by which laterality of treatment may be relevant for mental illness stimulation.

Traditional protocols for functional network visualization have applied the community detection algorithm to the cluster distribution of the group dataset to derive an average group‐representative network model (Akiki & Abdallah, 2019). This process has been shown to have several limitations, which may distort important network features, particularly when applied to highly heterogeneous patient datasets (Jeub et al., 2018). Recent work has validated a superior approach which first maps the cluster distribution of each individual's network before performing a meta‐reclustering analysis afterward (Jeub et al., 2018; Lancichinetti & Fortunato, 2012). This approach leads to a higher degree of similarity between individual network cluster distributions and the group‐representative model than the traditional method.

### 4.3 Anatomic specificity in TMS targeting

For most mental illness–related applications, the TMS target is the DLPFC, which is targeted using craniometric measurements from the location of the motor cortex (Perera et al., 2016). The Human Connectome Project and others have demonstrated that this large region comprises at least 14 distinct functional regions, which often show functional connectivity with different large‐scale brain networks. The specificity of functional connectivity anomalies we noted raises the possibility that this level of anatomic specificity is important to achieve consistent results.

**FIGURE 3:**
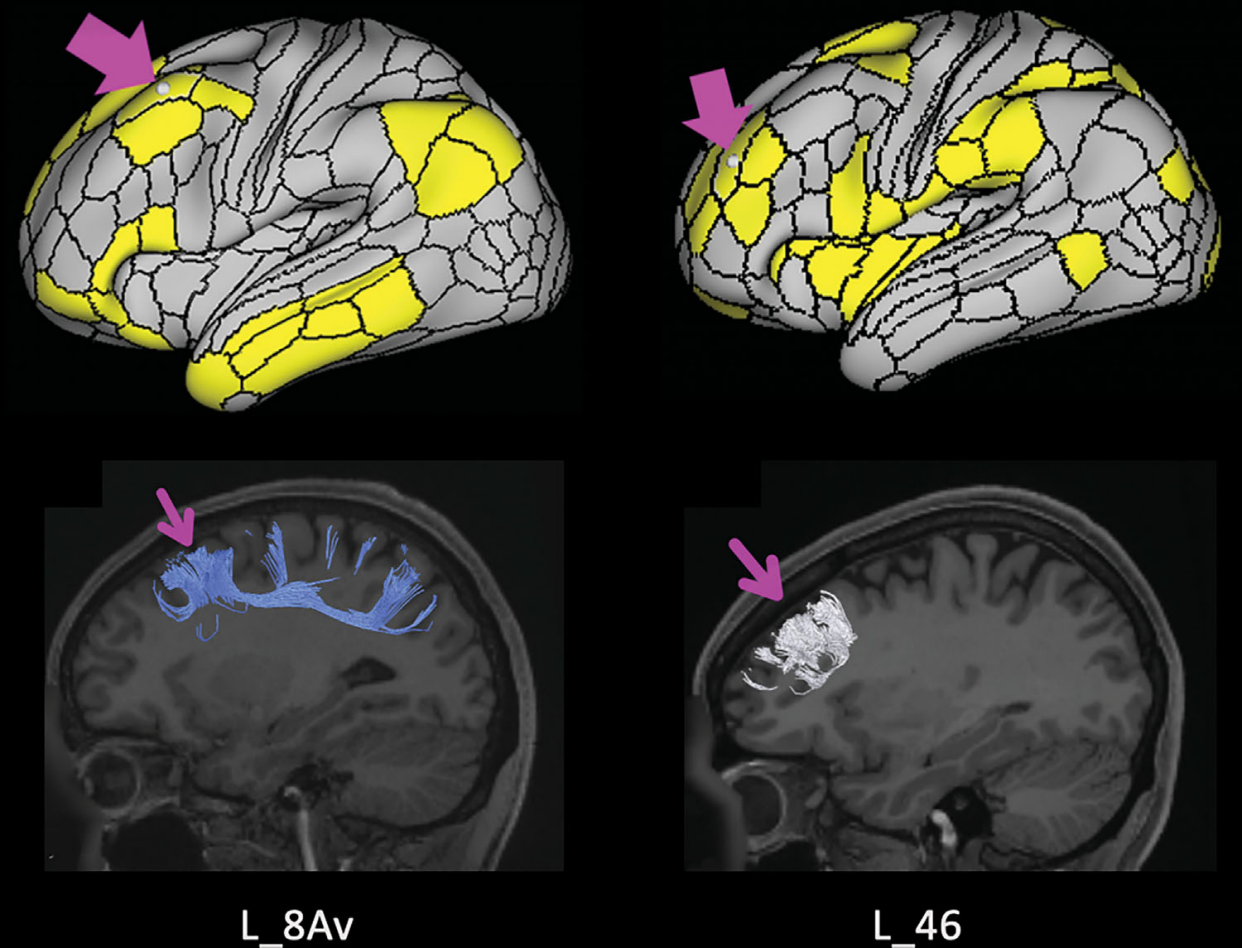
Anatomical location of areas left 8Av and 46. Demonstrates the similarities in location between left areas 46 and 8Av and the large differences in functional connections. Left area 46 has been suggested to be the target for depression treatment, area 8Av is immediately posterior to it, and is part of a different large‐scale brain network, and seems to be a promising anxiety target (Sandhu et al., 2021). Our results demonstrate consistent functional abnormalities associated with left area 8 Av, not left area 46, in our anxiety patients, which is consistent with previous literature which demonstrated that stimulation of an area similar to left 8 Av has been shown to mediate anxiosomatic symptoms, while stimulation of the region anterior to it, left 46, mediated dysphoric (Siddiqi et al., 2020).

One such important anatomic distinction involves area 46 and area 8Av, which are highlighted in Figure 3. Note that while area 46 has been suggested by some to be the target for depression treatment (Moreno‐Ortega et al., 2020), area 8Av is immediately posterior to it, and is part of a different large‐scale brain network, and also seems to be a promising anxiety target (Akiki & Abdallah, 2019). This has been demonstrated wherein stimulation of an area similar to 8Av has been shown to mediate anxiosomatic symptoms (e.g., irritability, decreased sexual interest, changes in sleep pattern), while stimulation of the region anterior to it, area 46 (part of the CEN) mediated dysphoric symptoms (e.g., anhedonia, guilt, psychomotor retardation) (Siddiqi et al., 2020). This suggests that it is possible that missing the target by 1 cm in the anterior‐posterior plane could be enough to stimulate the wrong network. In fact, such minuscule variations in target precision have recently been postulated to be the primary reason for the failure of a large RCT trial assessing TMS for treatment‐resistant MDD (Rosen et al., 2021). A recent study on veterans with treatment‐resistant MDD demonstrated that area 46 was the common target in the successful treatment responders to rTMS compared to area 8Av, which was the common target in nonresponders (Rosen et al., 2021; Yesavage et al., 2018). This is also supported by the improved and consistent results achieved by the individualized, image‐guided TMS utilized in our cohort.

### 4.4 Where we are and future directions: individualized TMS target selection

It is important to note that both individualized or connectome‐based TMS target selections are not new. Differences in functional connectivity have been previously correlated with differences in the clinical efficacy of TMS for patients with depression (Fox et al., 2012). Furthermore, methods accounting for individual differences, such as with PET scans or anatomy based MRI coordinates, are superior to standard craniometric TMS target selections, but have still produced variable results (Fitzgerald et al., 2009; Herwig et al., 2003). Importantly, many studies commonly utilize group‐average metrics (“group maps”) to guide TMS target selection and have produced results superior to those which targeted individualized foci (Fitzgerald, 2021; Fox et al., 2013; Herwig et al., 2003). This may be due to inherent limitations with the technology utilized, such as individual PET maps which may provide too much noise (Fox et al., 2013; Garcia‐Toro et al., 2006; Herwig et al., 2003). Fox et al. (2013) illustrated the feasibility of single‐subject, connectivity‐guided TMS targets within the left DLPFC in 2 patients with depression. Unfortunately, patients with GAD and depression, amongst many other mental illnesses, have significant individual variability in the connectivity of their affected brain networks, suggesting the need to replicate single‐subject, connectivity‐guided TMS selections in patients with GAD as well.

One study on patients with GAD demonstrated that a pretreatment provocation fMRI experiment (gambling) can determine active targets in individual subjects to guide the rTMS treatment, resulting in improved anxiety symptoms (Bystritsky et al., 2008, 2009). However, individualized, connectomic approaches based on rsfMRI data have not been described for patients with GAD as shown by Fox et al. (2013). Compared to task‐based fMRI, rsfMRI data do not require a task and instead focuses on spontaneous BOLD signal fluctuations, which is attractive in patients with psychiatric illness, more rapid for larger clinical trial executions, and also provide benefits for more data‐driven, machine learning approaches like the ones applied in the current study (Lv et al., 2018).

The current findings suggest the feasibility of an agile, connectomic approach at the single‐subject level based on rsfMRI data, which can target clinically effective TMS targets in a consistent and symptom‐specific manner. Applications in subsequent larger clinical trials will further demonstrate and optimize the clinical utility of this data‐driven approach for agile aTBS protocols.

### 4.5 Limitations

It is important to note that the presented research is made from our preliminary data as a proof‐of‐concept, and as such there are limitations that need to be considered when drawing conclusions from our work. The lack of control condition paired with the small cohort of presented data, limits the present findings to our data set. Further research should corroborate the findings of this study using a control condition and a larger participant pool to more rigorously understand the possible benefits of this connectomic approach, even though it may be logistically difficult to implement a control condition for subtype‐specific target selection. Additionally, our small cohort had variable presentations of anxiety, which will need to be considered in future research. To this end, we provided the subject‐level data to promote transparency for future researchers.

To maximize the understanding of our presented approach's effectiveness, future research should include a condition that uses sham stimulation with our presented targeting approach and an additional condition that uses active stimulation with the traditional targeting approach. Furthermore, this study is underpinned by the assumption that the cortical activity modifications by different forms of TBS likely also results in similar connectivity intensity modifications, wherein cTBS both decreases cortical activity and the associated connectivity that region has to other regions. Both this and the assumption that these stimulation protocols have net excitatory or inhibitory outputs have not been empirically confirmed. In saying that, our team believes this is a logical approach that did provide interesting and statistically significant clinical results, but we believe these assumptions are worthy of additional investigation.

Follow‐up procedures, including both posttreatment fMRI scans and long‐term rigorous metric collection, should be included in any future research, to understand if associated neural connectivity changes occur and if they correlate to objective improvements of anxiety. Particularly given the present studies inconsistent follow‐up response rates. Additionally, it is plausible that given the high rates of comorbidities within this study, that other attributable disease‐profiles, such as MDD diagnosis, are responsible for the vast improvement in outcomes which warrants additional research.

## 5 CONCLUSION

In our clinical experience, generalized anxiety patients have consistently displayed anomalies within the left 8Av and PGs region. Image‐guided TMS treatment targeted at these areas consistently produced improvements in anxiosomatic symptoms, and consequently patient quality of life. Our findings suggest that the DMN is the primary functional network disturbed in anxiety‐related disorders and that the disruption may be primarily left lateralized. The currently described work could inform the design of future studies, which should apply our data‐driven TMS target selection approach in a larger, controlled sample of patients, and should embolden the discovery of patient‐specific, connectomics‐informed real‐world clinical research.

## CONFLICT OF INTEREST STATEMENT

Isabella Young, Hugh Taylor, Peter Nicholas, Alana Mackenzie, Onur Tanglay, Karol Osipowicz, Stephane Doyen, and Michael Sughrue are employees of Omniscient Neurotechnology. Charles Teo, Stephane Doyen, and Michael Sughrue are board members. All other authors have no financial interests or potential conflicts of interest.

## PATIENT CONSENT

All patients provided informed consent at the time of treatment for their data to be used in retrospective analyses. All identifiable information has been omitted.

### PEER REVIEW

The peer review history for this article is available at https://publons.com/publon/10.1002/brb3.2914.

## Data Availability

All data used in analyses are presented in the current manuscript.
